# Frontoparietal Network Connectivity During an *N*-Back Task in Adults With Autism Spectrum Disorder

**DOI:** 10.3389/fpsyt.2020.551808

**Published:** 2020-09-09

**Authors:** Veronica Yuk, Charline Urbain, Evdokia Anagnostou, Margot J. Taylor

**Affiliations:** ^1^ Department of Diagnostic Imaging, The Hospital for Sick Children, Toronto, ON, Canada; ^2^ Neurosciences & Mental Health Program, SickKids Research Institute, The Hospital for Sick Children, Toronto, ON, Canada; ^3^ Department of Psychology, University of Toronto, Toronto, ON, Canada; ^4^ Neuropsychology and Functional Neuroimaging Research Group, Center for Research in Cognition & Neurosciences and ULB Neuroscience Institute, Université Libre de Bruxelles (ULB), Brussels, Belgium; ^5^ Laboratoire de Cartographie Fonctionnelle du Cerveau, Hôpital Erasme, Université Libre de Bruxelles, Brussels, Belgium; ^6^ Bloorview Research Institute, Holland Bloorview Kids Rehabilitation Hospital, Toronto, ON, Canada; ^7^ Department of Neurology, The Hospital for Sick Children, Toronto, ON, Canada; ^8^ Department of Paediatrics, University of Toronto, Toronto, ON, Canada; ^9^ Department of Medical Imaging, University of Toronto, Toronto, ON, Canada

**Keywords:** autism, connectivity, working memory, theta, alpha, maintenance, recognition, MEG

## Abstract

**Background:**

Short-term and working memory (STM and WM) deficits have been demonstrated in individuals with autism spectrum disorder (ASD) and may emerge through atypical functional activity and connectivity of the frontoparietal network, which exerts top-down control necessary for successful STM and WM processes. Little is known regarding the spectral properties of the frontoparietal network during STM or WM processes in ASD, although certain neural frequencies have been linked to specific neural mechanisms.

**Methods:**

We analysed magnetoencephalographic data from 39 control adults (26 males; 27.15 ± 5.91 years old) and 40 adults with ASD (26 males; 27.17 ± 6.27 years old) during a 1-back condition (STM) of an *n*-back task, and from a subset of this sample during a 2-back condition (WM). We performed seed-based connectivity analyses using regions of the frontoparietal network. Interregional synchrony in theta, alpha, and beta bands was assessed with the phase difference derivative and compared between groups during periods of maintenance and recognition.

**Results:**

During maintenance of newly presented vs. repeated stimuli, the two groups did not differ significantly in theta, alpha, or beta phase synchrony for either condition. Adults with ASD showed alpha-band synchrony in a network containing the right dorsolateral prefrontal cortex, bilateral inferior parietal lobules (IPL), and precuneus in both 1- and 2-back tasks, whereas controls demonstrated alpha-band synchrony in a sparser set of regions, including the left insula and IPL, in only the 1-back task. During recognition of repeated vs. newly presented stimuli, adults with ASD exhibited decreased theta-band connectivity compared to controls in a network with hubs in the right inferior frontal gyrus and left IPL in the 1-back condition. Whilst there were no group differences in connectivity in the 2-back condition, adults with ASD showed no frontoparietal network recruitment during recognition, whilst controls activated networks in the theta and beta bands.

**Conclusions:**

Our findings suggest that since adults with ASD performed well on the *n*-back task, their appropriate, but effortful recruitment of alpha-band mechanisms in the frontoparietal network to maintain items in STM and WM may compensate for atypical modulation of this network in the theta band to recognise previously presented items in STM.

## Introduction

Adults with autism spectrum disorder (ASD) demonstrate difficulties with a variety of executive functions (1–4), one of which is working memory (WM), which refers to the ability to hold and manipulate information in mind (5, 6). WM is related to short-term memory (STM), which involves the mental storage of information for a short period of time (7, 8), and which is also impaired in adults with ASD (9–12). Given their link with each other, and their influence on cognitive capabilities, such as intelligence and academic achievement (13–18), understanding the nuances and the extent of STM and WM impairments in ASD is a crucial first step in improving cognitive outcomes in this population. The current literature points to a more severe deficit in ASD in visual, especially visuospatial, aspects of STM and WM, rather than in verbal STM and WM (10, 19–23), though people with ASD exhibit impairments in both modalities (11, 24). Neuroimaging work has additionally shown that individuals with ASD exhibit atypical neural activity and connectivity during visual STM tasks (25–27) and in both visual and verbal WM tasks (28–32).

Several functional neuroimaging studies of STM and WM have demonstrated activation of a frontoparietal network (33–39) consisting mainly of the dorsolateral prefrontal cortex (dlPFC), which includes the superior and middle frontal gyri (SFG and MFG), and of the inferior parietal lobule (IPL). The frontoparietal network is thought to exercise cognitive control to adapt to rapidly changing goals and demands (40–44) that certainly occur in STM and WM tasks. The IPL has been primarily associated with maintenance (45–51), which entails the temporary storage of information in STM or WM, though the dlPFC has also been implicated (46, 48, 52–56). The dlPFC is additionally involved in recognition of previously presented or repeated stimuli (57–60), a function which encompasses access to or selection of relevant stimulus representations in STM or WM, and it also plays a role in updating or manipulation of information in WM. Much of the STM and WM literature in ASD has utilised paradigms tapping both maintenance and recognition processes; during such tasks, individuals with ASD show differential activation of this frontoparietal network across development, exhibiting increased activity in the dlPFC during childhood (27, 61), but the opposite in adulthood (26, 31, 62). Moreover, they show poor modulation of these frontoparietal regions with increasing cognitive load (30, 63, 64).

More recent work has examined not only the activation of regions in the frontoparietal network, but also how they communicate or synchronise with each other and with other brain areas. These connections are thought to be fundamental for exerting top-down control on other areas and networks for successful task performance (41, 42, 65–70). In the ASD population, studies generally demonstrate that areas in the frontoparietal network are less coupled with each other and with other regions of the brain (26, 29, 31). This reduced functional connectivity suggests impairments in integrating information amongst brain areas during maintenance and recognition, which may contribute to ASD symptomatology (25, 32). These findings echo the current literature on connectivity in ASD, which posits that individuals with ASD show decreased long-range functional connectivity and altered local connectivity across a range of contexts (71–76), suggesting that a deficit in neural communication may account for the cognitive difficulties observed in the ASD population.

Although neural long-range synchrony, especially in the theta and alpha frequency bands, has been linked to STM and WM maintenance and recognition processes (39, 77–85), the specific frequency band(s) in which these connectivity differences occur in individuals with ASD have been less explored. To our knowledge, only one study has demonstrated reduced alpha-band connectivity in children with ASD during a WM task, reflecting inefficient processing during recognition of repeated stimuli that was associated with severity of their ASD symptoms (32). Frequency-specific differences in STM- or WM-related connectivity in adults with ASD and their relation to behaviour have yet to be examined, even though these abilities can impact adaptive behaviours in individuals with ASD (3, 86, 87).

Thus, the present study investigated whether adults with ASD demonstrate connectivity differences when engaging STM and WM processes, and if they are frequency-dependent. Adults with and without ASD performed an *n*-back task, a classic paradigm in which participants view a series of stimuli and are asked to recall whether the current stimulus was also presented *n* trials earlier (88). We measured and compared connectivity between the control and ASD groups during maintenance and recognition of novel visual stimuli for both 1-back and 2-back versions of the task. The 1-back condition involves mainly STM processes, as an individual is only required to maintain and recognise the stimulus shown in the previous trial in mind. On the other hand, the 2-back condition elicits WM processes, as one must continuously monitor and update information stored in and retrieved from memory. Due to their involvement in STM (38, 39, 83, 84, 89–93) and WM mechanisms (77–79, 94–96) as well as long-range interregional communication (97–99), we specifically contrasted phase synchrony in the theta, alpha, and beta frequency bands. We also focused on connections amongst frontoparietal network regions and the rest of the brain, given its known role in STM (37–39, 84) and WM (33, 36, 100, 101), and since prior work has demonstrated deficits in this network in ASD (25, 27, 31). As individuals with ASD show deficits in tasks involving STM and WM maintenance (11, 20), decreased brain activity during STM recognition (27), and reduced connectivity during WM recognition (32), we predicted that adults with ASD would demonstrate decreased interregional connectivity during both maintenance and recognition of novel stimuli in STM and WM. We further hypothesised that these differences would appear in the alpha band, in line with our previous findings in children with ASD (32), and given its link with STM and WM processes, especially maintenance (77, 85, 102–105).

## Materials and Methods

### Participants

We recruited 92 adults aged 18–40 years, inclusive, for this study, approved by the Research Ethics Board at the Hospital for Sick Children. Individuals were included if they were not born prematurely, had no MRI or MEG contraindications, and demonstrated an IQ ≥ 70, measured using the full-scale, two-subtest version of the Wechsler Abbreviated Scale of Intelligence (WASI or WASI-II) (106, 107). Control adults were additionally screened for any developmental, neurological, or psychological disorders. Adults with ASD had a primary diagnosis of ASD by an experienced clinician, which was confirmed by the Autism Diagnostic Observation Schedule (ADOS-G or ADOS-2) (108, 109). All participants gave informed written consent before taking part in the study.

Participants were excluded if they performed poorly on the task (i.e., ≤50% accuracy on 1-back task or ≥50% false alarm rate), had a low number of correct trials (<40 in each condition) after accounting for artefacts, or poor data quality (e.g., poor head localization in the MEG). We then matched participants in the ASD group with those in the control group on age (within two years) and sex, and subsequently excluded any control participants who could not be matched. As participants tended to perform better on the 1-back than the 2-back version of the task, and as we evaluated the 1- and 2-back data separately, the final samples for these two analyses differed; the sample for the 2-back analysis was a subset of that for the 1-back analysis. For the 1-back task, 39 control adults (26 males; 27.15 ± 5.91 years old) and 40 adults with ASD (26 males; 27.17 ± 6.27 years old) met all inclusion and exclusion criteria. For the 2-back task, 29 control adults (19 males; 26.40 ± 5.79 years old) and 30 adults with ASD (19 males; 26.36 ± 6.26 years old) were included in the analyses. Neither sample differed significantly in age, sex, or IQ, and mean calibrated severity scores on the ADOS for both ASD samples were around 7 (**Table 1**).

**Table 1:** Demographics for 1-back and 2-back samples.

	Control	ASD	*t* or *X^2^*	*p*
	Mean (SD) or Count	Mean (SD) or Count		
***1-back***	*N* = 39	*N* = 40		
Age	27.15 (5.91)	27.17 (6.27)	0.02	0.99
Sex	26 M, 13 F	26 M, 14 F	2.43×10^-31^	1
IQ	114.34 (11.36)	111.79 (14.37)	0.86	0.39
ADOS CSS	—	6.89 (2.25)	—	—
***2-back***	*N* = 29	*N* = 30		
Age	26.40 (5.79)	26.36 (6.26)	0.03	0.98
Sex	19 M, 10 F	19 M, 11 F	6.01×10^-31^	1
IQ	115.45 (11.98)	113.48 (13.04)	0.60	0.55
ADOS CSS	—	7.00 (2.09)	—	—

ADOS, Autism Diagnostic Observation Schedule; CSS, calibrated severity score.

### Experimental Design

#### Questionnaires

To obtain a standardised measure of WM, we asked participants and their informants (e.g., partner or parent) to complete the Behavior Rating Inventory of Executive Function, Adult Version (BRIEF-A) (110). This questionnaire assesses difficulties with a variety of executive functions that an individual may experience in everyday life. It provides *t* scores reflecting the degree of impairment on a particular executive function scale, as well as composite scores. We used *t* scores on the WM scale of the BRIEF-A, with higher scores denoting more severe deficits in WM. Participants also filled out the Social Responsiveness Scale, Second Edition (SRS-2) (111). The Total *t* score was taken to gauge ASD symptom severity.

#### *N*-Back MEG Task

**Figure 1:**
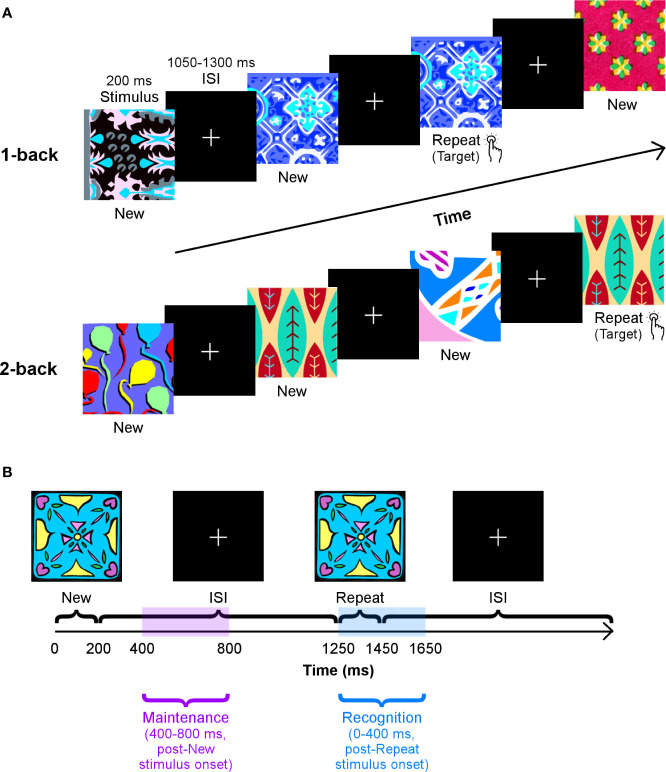
The *n*-back task. **(A)** Participants**’** performance on two loads of this task — 1-back (top row) and 2-back (bottom row) — were tested in separate blocks. They were instructed to press a button as quickly as possible when they recognised that a stimulus had been repeated one or two trials earlier. Images were presented for 200 ms, and the interstimulus interval varied between 1,050–1,300 ms. **(B)** A schematic of the time windows used to analyse working memory maintenance and recognition processes, with the 1-back load as an example.

Participants performed a visual *n*-back task with two loads, 1- and 2-back (**Figure 1A**), to elicit STM and WM processes. This task was used in our previous work examining differences in brain activation and functional connectivity between children with and without ASD (27, 32). Our task protocol was similar; stimuli consisted of novel colourful abstract images presented serially for 200 ms each on a black background. During the interstimulus interval, participants saw a white fixation cross for a random duration between 1,050–1,300 ms. Participants pressed a button if the most recently presented stimulus matched that shown *n* trials previously.

The two loads of the *n*-back task were run in separate blocks. The l-back load scenario consisted of 285 trials: 190 unique images were presented, and 95 of these were shown again on the subsequent trial. The 2-back load segment of the task contained 330 trials: 220 distinct images were shown, of which 110 were repeated two trials later. Stimuli in the 1- and 2-back loads did not overlap. We refer to trials in which stimuli are presented for the first time as **“**New,**”** and those in which they are shown again as **“**Repeat.**”**

All participants first practised both blocks of the task and were given feedback outside of the MEG scanner to ensure they understood the task requirements. Individuals viewed the task on a rear projection screen 80 cm away from the MEG dewar. *Presentation 18.1* software (Neurobehavioral Systems Inc., https://www.neurobs.com/presentation) was used to display the task, as well as record participant responses.

#### Neuroimaging Data Aquisition

A 151-channel CTF MEG system (Coquitlam, British Columbia, Canada) recorded MEG data at a 600 Hz sampling rate from participants during the task. Adults lay supine with their head in the MEG dewar. Head position was tracked continuously through three fiducial coils on the nasion and left and right pre-auricular points. To reduce noise in the data, an anti-aliasing low-pass filter at 150 Hz and a third order spatial gradient were applied.

A 12-channel head coil in a 3T MRI scanner (MAGNETOM, Siemens AG, Erlangen, Germany) recorded T1-weighted MRI data from participants. A sagittal 3D MPRAGE sequence (TR/TE = 2,300/2.96 ms, FA = 9°, FOV = 192×240×256 mm, voxel size = 1.0 mm isotropic) was utilised. Participants were scanned with radio-opaque markers at the MEG fiducial points to allow for coregistration of functional MEG data with structural MRI data.

### Statistical Analysis

#### Behavioural Data

WM capability in everyday life was compared between adults with and without ASD in the 1- and 2-back samples by examining whether there were group (control vs. ASD) or rater (self vs. informant) effects, as well as an interaction between the two, on the WM scale of the BRIEF-A. Performance on the *n*-back task was contrasted between groups by assessing the effect of group (control vs. ASD) on accuracy and median response time (RT) for the 1- and 2-back loads independently. Accuracy was assessed using d-prime (d′); hits were correct Repeat trials, and false alarms were incorrect New trials.

We used linear mixed effects models to investigate the effects on BRIEF-A data and *t* tests for the task performance measures. Analyses were carried out separately for the 1-back and 2-back samples in R 3.5.0 (R Core Team, https://www.r-project.org/). Significant results are reported for *p* < 0.05.

#### MEG Data

##### Preprocessing

MEG data preprocessing and analyses were done using the FieldTrip toolbox (112) in MATLAB 2017b (The MathWorks, www.mathworks.com/products/matlab/). Data were epoched from −1,500–2,000 ms, relative to stimulus onset. Signals were then filtered from 1–150 Hz with a fourth-order Butterworth bandpass filter, with 60 and 120 Hz notch filters. Artefacts from physiological sources (e.g., eyes and heart) were detected and removed with independent component analysis. Trials in which the signal was >2,000 fT or head movement was >5 mm were excluded. Of the remaining trials, only correct New and Repeat trials were used for further analyses.

Forward models based on the single-shell method (113) were created from each participant’s T1-weighted MRI data. Inverse models were constructed using the forward model and constrained to the centroids of the 90 regions of the Automated Anatomic Labeling (AAL) atlas (114). Activity at each centroid was taken to represent that respective AAL region. Time series at these sources were estimated using a linearly constrained minimum variance beamformer (115). The covariance matrix was computed on the MEG signal from −400–800 ms, to which 5% regularisation was applied. The neural activity index was calculated to ensure attenuation of centre-of-head noise biases.

##### Connectivity

As our task involved strong visual and motor responses, we performed seed-based analyses to focus on connectivity between core regions of the frontoparietal network and the rest of the brain to assess phase synchrony directly related to maintenance and recognition of novel visual stimuli. Therefore, we examined the connections amongst six bilateral regions of interest (ROIs) from the AAL atlas, as well as their links to the other AAL regions (except Heschl’s gyrus and olfactory cortex, as their roles in audition and olfaction are not involved in our task). Our ROIs were chosen based on meta-analyses of *n*-back studies (33, 35, 36). They consisted of the superior frontal gyri [SFG; (-19, 35, 42) and (20, 31, 44)], medial superior frontal gyri [mSFG; (-6, 49, 31) and (8, 51, 30)], middle frontal gyri [MFG; (-34, 33, 35) and (37, 33, 34)], inferior frontal gyri [IFG; (-47, 30, 14) and (49, 30, 14)], insulae [(-36, 7, 3) and (38, 6, 2)], and inferior parietal lobules [IPL; (-44, -46, 47) and (45, -46, 50)].

Connectivity between each pair of sources was quantified using the phase difference derivative (PDD) (116). Source-estimated data were filtered into our frequency bands of interest with Hamming-windowed FIR bandpass filters at the following passbands: 4–7 Hz (theta), 8–14 Hz (alpha), and 15–30 Hz (beta). The lower and upper stopband frequencies for each filter were at 0.6 and 1.9 times the lower and upper frequency cutoffs of each passband, respectively. To reduce detection of spurious connections due to signal leakage, filtered data were subsequently orthogonalized. The instantaneous phase for each source timeseries in each frequency band was obtained with the Hilbert transform. PDD values were calculated at each time point from −400–800 ms using the method outlined by Tewarie and colleagues (117).

Interregional neural communication during maintenance and recognition of novel visual stimuli was determined by considering phase synchrony values in two time windows: 400–800 ms, after the onset of a New stimulus, and 0–400 ms, following the presentation of a Repeat stimulus, respectively (**Figure 1B**). These windows were established based on average median RTs in both groups, which ranged from ~425–525 ms, across both loads (see Results section). Regarding recognition, we examined a window from 0–400 ms, post-Repeat stimulus onset and just before the lower end of the average median RT, as it would encompass processing related to successful recognition during correct Repeat trials. We compared phase synchrony in this window to that in a similar window of 0–400 ms, post-New stimulus onset (Repeat > New, 0–400 ms, poststimulus onset), as New trials act as a control condition that involves the first occurrence of the stimulus. To investigate maintenance, we evaluated a window from 400–800 ms, post-New stimulus onset, to prevent capturing any perceptual, encoding, and/or identification functions that may occur during early visual processing. Furthermore, we chose this interval to avoid overlap with our recognition analysis and the baseline window. We contrasted connectivity in this time window in New trials with connectivity in an equivalent window of 400–800 ms, post-Repeat stimulus onset in Repeat trials (New > Repeat, 400–800 ms, poststimulus onset). Mean connectivity at all pairwise connections for each participant and each condition in these comparisons was obtained by standardising PDD values in these windows by the baseline period, –400–0 ms, then averaging the resultant *z* scores over the entire time window of interest.

Statistical comparisons of within-group connectivity during maintenance and recognition were conducted as described above for both adults with and without ASD. We then tested for statistically significant group differences in each of these scenarios (e.g., Control vs. ASD, New > Repeat for maintenance; Control vs. ASD, Repeat > New for recognition). All within- and between-group comparisons were performed for the 1- and 2-back samples separately. For both types of contrasts, we performed cluster-based permutation testing, as implemented in the Network-Based Statistic toolbox (118), to find networks demonstrating significant differences between conditions and groups. Essentially, the Network-Based Statistic approach begins by performing *t* tests at each connection and applying a threshold, which we chose to be *t* = 2.641 (1-back) or *t* = 2.665 (2-back), which are equivalent to *p* < 0.005 in their respective samples. The robustness of the largest contiguous network formed from the suprathreshold connections was assessed with permutation testing. A null distribution of maximal network size was obtained by rearranging group labels over 5,000 permutations. This procedure allowed for the calculation of a family-wise error-corrected *p* value (*p*_FWE_) of the observed network. Networks were considered significant at *p*_FWE_ < 0.05. We used BrainNet Viewer (119) and code provided by Koelewijn and colleagues (120) to visualise these networks.

#### Brain-Behaviour Relations

We explored whether mean network connectivity in any of our group comparisons was associated with WM abilities as measured by the BRIEF-A, task performance (accuracy and median RT), and with ASD symptom severity. Thus, for any networks that differed significantly between groups, we performed regressions of each the BRIEF-A WM scale scores, d′, median RT, and SRS-2 Total scores on mean PDD values in those networks. We report any significant main effects of mean connectivity and/or its interaction with group for *p* < 0.05.

## Results

### Behaviour

**Figure 2:**
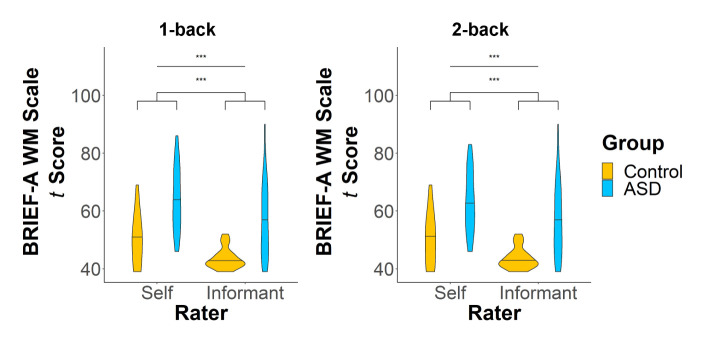
*T* scores on the working memory (WM) scale of the BRIEF-A for the 1-back (left panel) and 2-back (right panel) samples. There were significant main effects of both group and rater on WM scores. ***p < 0.001.

On the WM subscale of the BRIEF-A, adults with ASD demonstrated significantly more WM difficulties than controls (**Figure 2**) in both the 1-back (*F*(1,43) = 25.56, *p* < 0.0001, *d* = 0.65) and 2-back (*F*(1,38) = 22.18, *p* < 0.0001, *d* = 0.64) samples. Adults with ASD, compared to their informants, generally rated themselves higher on the WM scale (1-back: *F*(1,43) = 19.38, *p* = 0.0001, *d* = 0.34; 2-back: *F*(1,38) = 14.06, *p* = 0.0006, *d* = 0.32), indicating a greater number of difficulties with WM.

**Figure 3:**
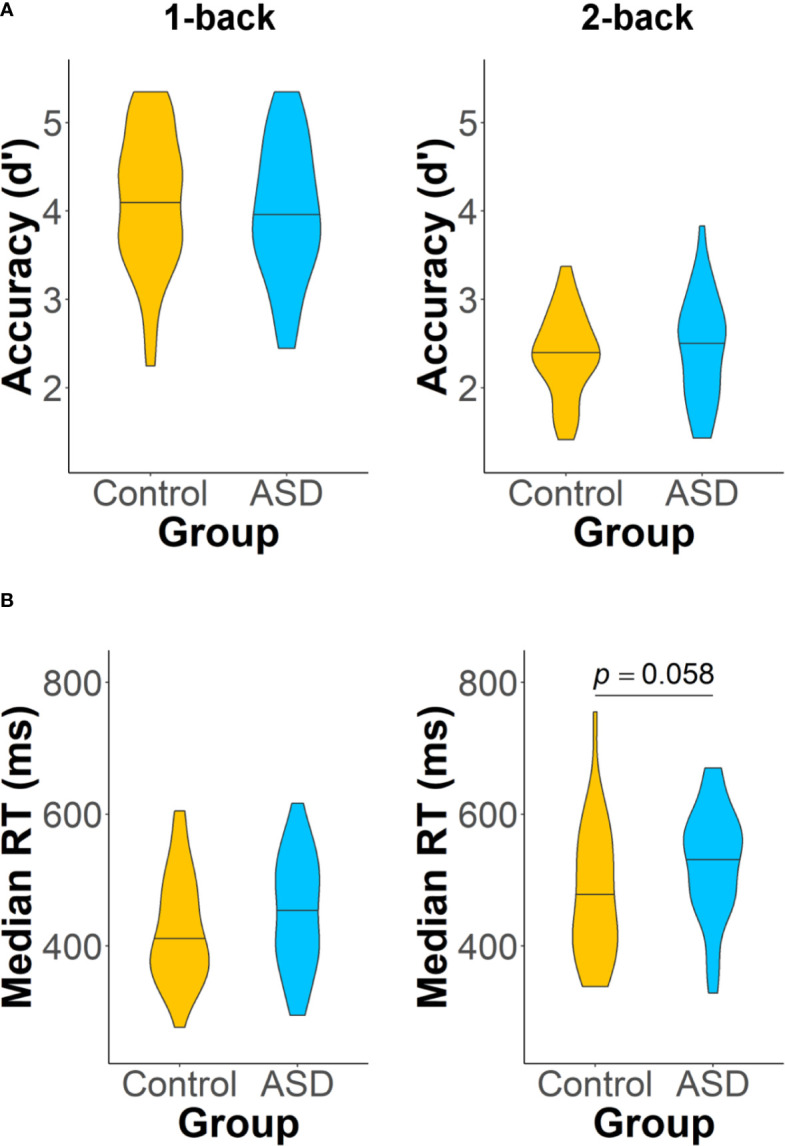
Accuracy **(A)** and median RT **(B)** on the two loads of the *n*-back task: 1-back (left panel) and 2-back (right panel). Adults with autism spectrum disorder (ASD) showed a trend of having longer median RTs than control adults on the 2-back task (*p* = 0.058). Analyses of all other task performance measures did not reveal any significant group differences.

On the *n*-back task, there were no group differences in accuracy (1-back: *t*(76.93) = 0.77, *p* = 0.44, *d* = 0.17; 2-back: *t*(55.30) = 0.51, *p* = 0.61, *d* = 0.13) in the 1- or 2-back loads (**Figure 3A**). The two groups also had similar median RTs in the 1-back load (*t*(76.97) = 1.51, *p* = 0.13, *d* = 0.34), but their differences in median RT during the 2-back load approached significance (*t*(54.71) = 1.93, *p* = 0.058, *d* = 0.50), such that adults with ASD had slightly longer median RTs than controls (**Figure 3B**).

### Neuroimaging

#### Maintenance

**Figure 4:**
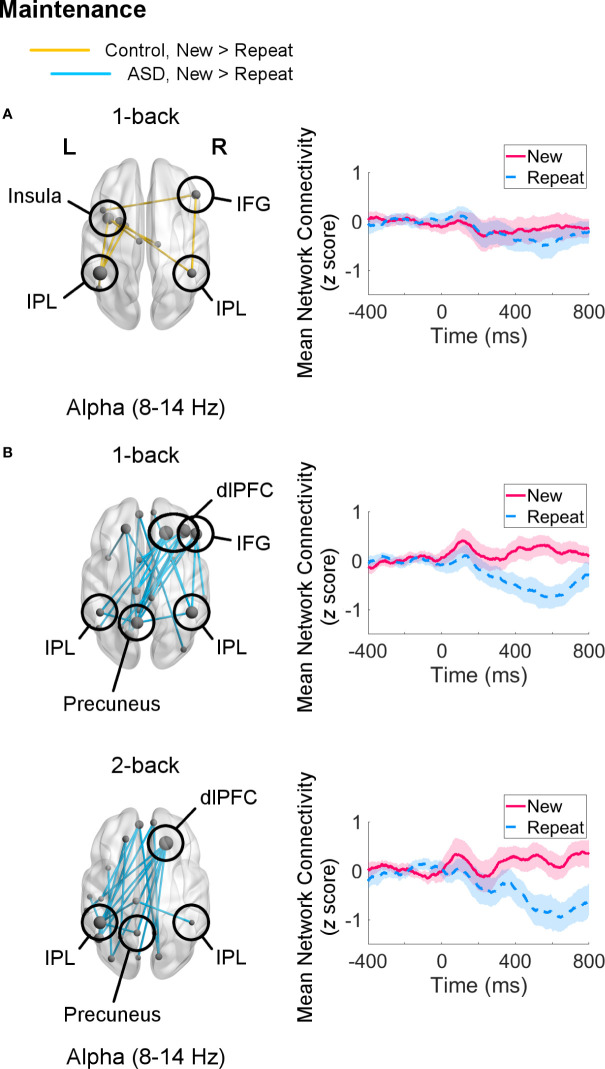
Networks showing increased connectivity during maintenance of novel visual stimuli (New versus Repeat trials, 400–800 ms, poststimulus onset) during the 1-back and 2-back loads (left) and mean connectivity of these networks between −400–800 ms (right). Node size is scaled by number of connections. Connectivity values are given as *z* scores. **(A)** Control adults showed recruitment of a network in the alpha band in the 1-back load (*p*_FWE_ = 0.048), but not in the 2-back load. **(B)** Adults with autism spectrum disorder (ASD) displayed greater connectivity in a network in the alpha band similar between the 1-back (*p*_FWE_ = 0.001) and 2-back (*p*_FWE_ = 0.002) samples.

For the 1-back task, during the maintenance window, both control adults (*p*_FWE_ = 0.048) and adults with ASD (*p*_FWE_ = 0.001) recruited aspects of the frontoparietal network selectively in the alpha band. In the control group, the network hub with the most (four) connections was the left IPL, which mainly communicated with other left hemisphere regions, such as the left insula (**Figure 4A**). The right IPL and IFG were also involved in this network, though they were each only connected to two other regions. In the ASD group, both the right SFG and MFG were the main hubs with five connections each, linking the right dlPFC with the right IPL and with several left posterior regions, including the left IPL and precuneus (**Figure 4B**). In this network, the right IFG also showed synchrony with the right IPL and precuneus.

For the 2-back load, adults with ASD continued to show increased alpha-band connectivity during New versus Repeat trials (*p*_FWE_ = 0.002) in a right dlPFC-left posterior network linking the right SFG hub with the left IPL and precuneus (**Figure 4B**). Control adults did not exhibit greater engagement of any networks in any frequency band for New compared to Repeat trials for this load. There were no significant group differences in the maintenance interval for either load.

#### Recognition

**Figure 5:**
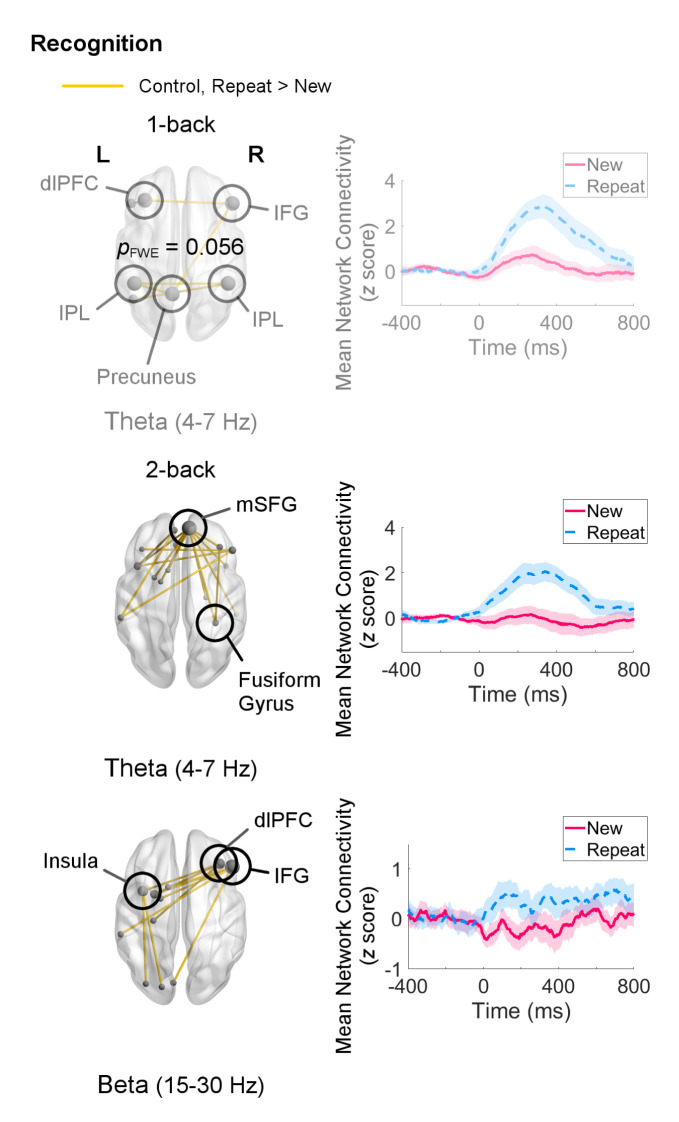
Networks showing increased connectivity during recognition of repeated visual stimuli (Repeat versus New trials, 0–400 ms, poststimulus onset) for the 1-back and 2-back loads in the control group (left) and mean connectivity of these networks between −400–800 ms (right). Node size is scaled by number of connections. Connectivity values are given as *z* scores. In the 1-back load, control adults recruited a theta-band network, but it was only significant at a trend level (*p*_FWE_ = 0.056). In the 2-back load, they exhibited greater connectivity in networks in the theta (*p*_FWE_ = 0.0084) and beta (*p*_FWE_ = 0.015) bands. Adults with autism spectrum disorder (ASD) did not show differential connectivity during recognition.

**Figure 6:**
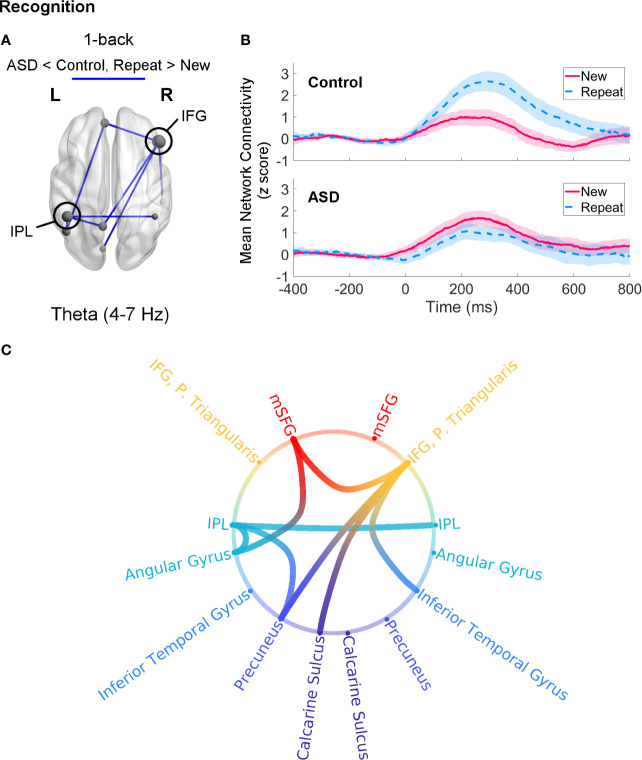
Theta-band connectivity in adults with autism spectrum disorder (ASD) compared to controls during recognition of novel visual stimuli (Repeat versus New trials, 0–400 ms, poststimulus onset) in the 1-back load. **(A)** Adults with ASD showed significantly reduced (*p*_FWE_ = 0.046) theta-band connectivity in a network with hubs in the right IFG and left IPL. Node size is scaled by number of connections. **(B)** Mean connectivity in this network between −400–800 ms in the control (top) and ASD (bottom) groups. Connectivity values are given as *z* scores. **(C)** Network connectivity represented as a circle plot. Nodes are colour-coded in the following manner, from top to bottom: medial frontal structures (red), frontal areas (orange), parietal regions (turquoise), temporal areas (blue), medial parietal structures (dark blue), occipital areas (purple).

Recognition processes in the 1-back load were associated with a trend (*p*_FWE_ = 0.056) increase in theta-band network connectivity in control adults for Repeat relative to New trials. This network was sparse, consisting mainly of a few connections (two each) amongst the left MFG, right IFG, bilateral IPL, and precuneus (**Figure 5**). Adults with ASD did not show any differential connectivity for Repeat versus New trials in any frequency band. When comparing the two groups, adults with ASD exhibited significantly decreased theta-band connectivity compared to control adults (*p*_FWE_ = 0.046) in a network of regions in which the right IFG and left IPL were major hubs (**Figure 6**).

Within-group analyses for the 2-back load during recognition revealed organisation of networks in the theta (*p*_FWE_ = 0.0084) and beta (*p*_FWE_ = 0.015) bands in the control group (**Figure 5**). The theta-band network primarily involved coordination between the mSFG and temporal regions, for example the right fusiform gyrus. The beta-band network displayed a different topography, with the right IFG having the highest (five) number of connections, followed by the right MFG and left insula (three each). Although adults with ASD in this sample also did not show any differential connectivity for this analysis in any frequency band, no significant group differences were found for the 2-back load.

### Brain-Behaviour Relations

Mean connectivity in the theta-band network that differed between groups was not associated with any of our behavioural or clinical measures, nor was its interaction with group (all *p*s > 0.05).

## Discussion

Our study illustrates the complex distinctions in STM and WM processing between adults with and without ASD on both the behavioural and neural level. In our sample, adults with ASD performed equally as well as control adults on our visual *n*-back task, although there was a tendency for adults with ASD to have longer RTs in the 2-back block, during WM processing. This pattern was also observed by Lever and colleagues (121), who similarly demonstrated that despite being as accurate as controls on an *n*-back task, adults with ASD took significantly longer to respond. Slower RTs may be indicative of slower processing speed, which has also been reported in ASD ((122–127); but see (128, 129)). Whilst it may not affect performance on simple experimental STM or WM tasks, this trend towards longer responses or processing could have more noticeable effects in complex, everyday behaviours. Since deficits in processing speed and WM have been found in individuals with ASD (19, 86, 130–132), further work is needed to clarify the link between processing speed and WM abilities in ASD, especially as our ASD group reported WM difficulties on the BRIEF-A. Our neuroimaging analyses examined the underlying neural differences in the frontoparietal network responsible for maintenance and recognition of novel visual stimuli that may contribute to these impairments.

### Maintenance

During maintenance of novel visual stimuli, we observed that whilst adults with and without ASD did not differ significantly from each other, they exhibited distinct topologies and sizes of the networks they recruited. During the 1-back task, the alpha-band network recruited by controls was fairly left-lateralized, as the left IPL and left insula showed the most connections. The IPL is an integral part of the frontoparietal network involved in *n*-back tasks (33, 35, 36), and it may serve to maintain stimulus information in STM and WM (37, 45–48, 133–136), as well as shift attention to specific items in WM (137, 138). Although the IPL is more commonly associated with spatial STM and WM (e.g., (49, 139–145)), there is evidence that it similarly participates in object or image identity STM and WM (33, 36, 37, 50, 146–149). The insula has also been associated with object STM capacity (150), but its principal function is in recruiting the frontoparietal network when attentional and executive resources are needed (151–153) through its functional connections with the dlPFC (154, 155).

In comparison, adults with ASD demonstrated greater alpha-band interregional synchrony for New versus Repeat trials during the 1- and 2-back loads. The networks recruited in both loads were similar; they had a right frontal-to-left parietal configuration and included the right dlPFC, bilateral IPLs, and precuneus. The dlPFC is a key region in STM and WM processing, employing top-down control to maintain, monitor or update, and manipulate task-relevant information in mind (52, 53, 56, 156–159), by focusing attention to target stimulus representations in the IPL (54, 101, 160, 161). The precuneus mediates several higher-order cognitive functions (162–164), and given its connections with the IPL, it is likely involved in visuospatial processing (165–167) and visual recall (168–170) in this task. The particular involvement of the precuneus in the ASD group may reflect greater mental engagement, as our previous work showed that children with ASD activated the precuneus more with heavier cognitive load (27). Whilst we did not detect any significant group differences, the recruitment of additional STM and WM regions — the dlPFC and precuneus — in ASD group compared to controls, as well as of several other brain regions, may reflect effortful maintenance processes in ASD adults.

The particular arrangement of this right frontal, left posterior network not only mirrors previous work finding atypical functional lateralization in ASD during a WM task (31), but also suggests that maintenance of novel visual information is challenging for adults with ASD. In control adults, increasing task load has been associated with bilateral activation of the IPL (171, 172) and dlPFC (34, 173, 174), with a few demonstrating stronger effects in right dlPFC (175–177). Greater dlPFC-IPL (frontoparietal) connectivity has also been linked to higher task load (77, 178). Therefore, the strong involvement of the right dlPFC and its connection to the left IPL in the ASD group in both the 1- and 2-back loads indicates that holding visual stimuli in mind is mentally taxing for adults with ASD. In both groups, however, brain regions synchronised selectively in the alpha band, which has been implicated in STM and WM maintenance (77, 84, 85, 91, 103, 105, 179). Thus, our findings demonstrate that adults with ASD utilise appropriate neural mechanisms to successfully maintain novel visual stimuli in STM and WM, but it may be effortful for them.

### Recognition

During recognition of repeated visual stimuli, adults with ASD exhibited no differential connectivity between Repeat and New trials for either the 1- or 2-back load. Hence, when contrasted with the control group, they showed significantly decreased theta-band synchrony compared to controls during the 1-back load in a network with hubs in the right IFG and left IPL. The ventrolateral PFC, which includes the IFG, is thought to work with the IPL for active retrieval of information (53). Specifically, the right IFG plays a major role in inhibition (180–184) and potentially preventing proactive interference during WM (137, 185–187), whereas the IPL, in addition to storing stimulus representations, may be responsible for retrieval (146). Since the selection of relevant information in STM during recognition may not only involve enhancement of target stimulus representations, but also suppression of irrelevant ones, and given evidence that individuals with ASD experience deficits in interference control ((188–190); but see (191, 192)), the reduced involvement of the IFG and IPL during recognition may reflect a potential breakdown in regulating interference from other stimuli in STM. This possible deficit in inhibiting task-irrelevant stimuli is further corroborated by the fact that these differences occurred in the theta band, as interregional theta-band connectivity is thought to mediate long-range neural communication, top-down control, and integration of distant regions (99, 193–195), especially during retrieval (196).

A similar effect was not found when contrasting the two groups in the 2-back task, which may be attributable to the slightly smaller sample size and therefore less power due to a greater variation in response in this condition. Importantly, the control group showed recruitment of theta-band networks for both loads (and additionally a beta-band network in the 2-back load), whereas the ASD group showed no greater connectivity in either load for Repeat compared to New trials. As the pattern of within-group results was similar across loads, a comparable but subthreshold trend may exist in the 2-back condition.

Taking into account that decreased connectivity has been observed in fMRI in ASD during other *n*-back tasks (25, 29, 31), our findings in the 1-back condition substantiate reports of long-range underconnectivity in ASD (72, 74, 197–199). The specificity of this difference to the theta band is also in line with prior work emphasising the role of theta oscillations in STM (39, 82, 84) and recognition/retrieval (196, 200–202). This result is in contrast to our previous work (32), where we found that these differences occurred in the alpha band during recognition of repeated visual stimuli in a WM task. However, there are some key distinctions between these two studies. First, the present work observed that connectivity in the fronto*parietal* network was only significantly reduced in the ASD relative to control group in the theta band during the 1-back condition, whilst we previously reported decreased alpha-band synchrony in a fronto*temporal* network in individuals with ASD during the 2-back condition (32). Therefore, it is unclear from our prior work whether frontoparietal network connectivity also differed between individuals with and without ASD in the 2-back condition in either the theta or alpha bands, and whether frontotemporal network connectivity was affected in the current study. Second, our earlier study assessed children with ASD, whereas here we included only adults with ASD; thus, part of this discrepancy may be attributable to maturational processes. As both theta and alpha bands have been associated with STM and WM functions (77, 82–85, 95, 196, 203–205), it may be that connectivity related to recognition strengthens in the alpha band but weakens in the theta band over development in ASD. Whilst global network efficiency in the theta and alpha bands increases with age (206), these longitudinal changes in frequency-specific, long-range neural connectivity have not been characterised in ASD, neither has the frontoparietal network or any other networks related to STM or WM explicitly. Future work into the developmental trajectory of the spectral component of the frontoparietal network and its relation to STM and WM in ASD will be necessary to clarify these distinct findings in children and adults with ASD.

Another important consideration is that mean connectivity in the theta-band network that differed between groups in the 1-back load was not correlated with our behavioural measures. However, as our analyses probed very specific STM (1-back) and WM (2-back) mechanisms, it may be challenging to relate these fine neural differences to overall task performance and more complex behaviours drawing on WM abilities in everyday life. Therefore, it will be valuable for prospective work to evaluate whether these findings persist in more ecologically valid tasks of STM and WM.

### Conclusion

Our neuroimaging study revealed unique aspects of STM and WM maintenance and recognition processes in adults with ASD. We demonstrated that whilst adults with ASD appropriately employ alpha-band oscillatory mechanisms to facilitate maintenance of novel visual stimuli in STM and WM, the distinct topology and extent of the recruited networks suggest that these functions are effortful for individuals with ASD. The strong engagement of maintenance processes may offset the observed atypicalities in theta-band connectivity in the ASD group during recognition of previously presented visual stimuli, at least in scenarios tapping STM. Given the spatial and spectral specificity of our findings, we propose that alpha-band connectivity between the dlPFC and IPL in the frontoparietal network enhances the neural representations of target stimuli during maintenance, thereby countering potentially stronger interference effects that occur during recognition due to reduced theta-band synchrony of the IFG and IPL with other regions of the brain.

We are the only study to date to use MEG to detail these maintenance and recognition processes and their spectral properties in the frontoparietal network in ASD. Thus, additional work is needed to independently validate our findings and interpretations in other investigations of STM and WM functions, especially as the distinct connectivity patterns in the control and ASD groups whilst maintaining novel visual stimuli in STM and WM showed only qualitative differences. It would be important to examine maintenance and recognition in other STM and WM tasks that more clearly separate these processes, as in higher loads of *n*-back tasks, participants are required to maintain stimuli from previous trials in WM, even after recognising a repeated stimulus. This constraint may explain why we did not observe differential connectivity in the control group during the maintenance window of the 2-back load when comparing phase synchrony during New and Repeat trials, and perhaps why we did not find any significant group differences in networks recruited for maintenance of novel visual stimuli. Furthermore, since our paradigm only included 1- and 2-back loads, we were unable to robustly assess whether adults with and without ASD show discrepancies in network recruitment with increasing cognitive load. As previous work has suggested that individuals with ASD do not show load-dependent modulation of activity in the frontoparietal network (26, 30, 63, 207), the effect of load on network connectivity in ASD is an important consideration. Finally, future work should explore STM- and WM-related connectivity patterns in broader samples of individuals with ASD, as our study included mainly higher-functioning adults with ASD who were able to perform well on our *n*-back task (though who still reported difficulties with WM in everyday life), and since there may be considerable heterogeneity in network recruitment in the ASD population, which may account for the lack of differential connectivity at the group level during recognition in our ASD sample. Our findings demonstrate, however, atypical frontoparietal network connectivity in adults with ASD when engaging in recognition of repeated visual stimuli, especially in STM, and further research will be essential to uncovering the nuances of these discrepancies.

## Data Availability Statement

The raw data supporting the conclusions of this article will be made available by the authors, without undue reservation.

## Ethics Statement

The studies involving human participants were reviewed and approved by the Hospital for Sick Children Research Ethics Board. The patients/participants provided their written informed consent to participate in this study.

## Author Contributions 

MT conceived and designed the study. VY collected and analysed the data, interpreted the findings, and drafted the manuscript. CU, EA and MT contributed significantly to revising the manuscript. All authors contributed to the article and approved the submitted version.

## Funding

This work was funded by Canadian Institutes of Health Research grants MOP-119541 and MOP-142379 to MT and a Frederick Banting and Charles Best Canada Graduate Scholarships Doctoral Award awarded to VY.

## Conflict of Interest 

The authors declare that the research was conducted in the absence of any commercial or financial relationships that could be construed as a potential conflict of interest.
